# Promoter Hypermethylation of KLF4 Inactivates Its Tumor Suppressor Function in Cervical Carcinogenesis

**DOI:** 10.1371/journal.pone.0088827

**Published:** 2014-02-14

**Authors:** Wen-Ting Yang, Peng-Sheng Zheng

**Affiliations:** 1 Department of Reproductive Medicine, First Affiliated Hospital Medical School of Xi’an Jiaotong University, Xi’an, The People’s Republic of China; 2 Department of Biochemistry and Molecular Biology, Medical School of Xi’an Jiaotong University, Xi’an, The People’s Republic of China; 3 Section of Cancer Stem Cell Research, Key Laboratory of Environment and Genes Related to Diseases, Ministry of Education of People's Republic of China, Xi’an, The People’s Republic of China; Sapporo Medical University, Japan

## Abstract

**Objective:**

The KLF4 gene has been shown to be inactivated in cervical carcinogenesis as a tumor suppressor. However, the mechanism of KLF4 silencing in cervical carcinomas has not yet been identified. DNA methylation plays a key role in stable suppression of gene expression.

**Methods:**

The methylation status of the KLF4 promoter CpG islands was analyzed by bisulfite sequencing (BSQ) in tissues of normal cervix and cervical cancer. KLF4 gene expression was detected by RT-PCR, immunohistochemistry and western blot. KLF4 promoter methylation in cervical cancer cell line was determined by BSQ and methylation-specific polymerase chain reaction (MS-PCR). Cell proliferation ability was detected by cell growth curve and MTT assay.

**Results:**

The methylated allele was found in 41.90% of 24 cervical cancer tissues but only in 11.11% of 11 normal cervix tissues (P<0.005). KLF4 mRNA levels were significantly reduced in cervical cancer tissues compared with normal cervix tissues (P<0.01) and KLF4 mRNA expression showed a significant negative correlation with the promoter hypermethylation (r = −0.486, P = 0.003). Cervical cancer cell lines also showed a significant negative correlation between KLF4 expression and hypermethylation. After treatment with the demethylating agent 5-Azacytidine (5-Aza), the expression of KLF4 in the cervical cancer cell lines at both mRNA and protein levels was drastically increased, the cell proliferation ability was inhibited and the chemosensitivity for cisplatin was significantly increased.

**Conclusion:**

KLF4 gene is inactivated by methylation-induced silencing mechanisms in a large subset of cervical carcinomas and KLF4 promoter hypermethylation inactivates the gene’s function as a tumor suppressor in cervical carcinogenesis.

## Introduction

Cervical cancer is a major contributor to cancer-related death in females worldwide and accounts for 250,000 deaths each year [1]. Although infection with high-risk human papillomaviruses (HPV) is intimately related to the development of cervical carcinoma, progressing from an HPV-positive premalignant lesion to invasive carcinoma is a rare event [2], [3]. Several reports have suggested that the aggressive nature of human cervical carcinoma is related to a number of molecular abnormalities, including inactivation of various tumor suppressor genes and activation of various oncogenes [4]. The development of novel targeted therapies for cervical cancer has been hindered by the lack of sufficient genetic and epigenetic data concerning its pathogenesis and the paucity of targets [5], [6], [7], [8].

The KLF4 gene, a critical transcription regulator of cell growth and differentiation, has been reported to be dysregulated in several human cancers. The KLF4 gene was found to be frequently downregulated in gastric cancers, pancreatic ductal carcinoma, lung cancer, and medulloblastoma [9], [10], [11], [12]. Moreover, forced overexpression of KLF4 inhibits cell proliferation and growth of colon, bladder, and esophageal cancers [13], [14], [15]. However, KLF4 expression was shown to be increased in breast cancer and head and neck squamous cell carcinomas [16], [17]. The KLF4 gene was shown to be genetically and epigenetically inactivated in human pancreatic cancer and gastric cancer, as well as in medulloblastoma, and to be mutated in colon cancer [12], [18], [19], [20]. In our pervious study, the KLF4 gene was found to be inactivated and to function as a tumor suppressor in cervical carcinogenesis [21]. However, it remains unknown how KLF4 is silenced in cervical carcinomas.

In the present study, the methylation of some CpG islands in the KLF4 promoter was demonstrated in a large subset of cervical cancers, and this methylation was negatively correlated with protein expression. Restoring KLF4 expression by treating the cells with the demethylating agent 5-Aza inhibited the proliferation of SiHa and C33A cells. Our results support the hypothesis that KLF4 promoter methylation inactivates the gene’s function as a tumor suppressor in cervical carcinogenesis.

## Materials and Methods

### Study Subjects and Ethics Statement

24 patients were newly diagnosed with histologically confirmed and previously untreated (no radiotherapy or chemotherapy) primary cervical cancer from the First Affiliated Hospital of Xi’an Jiaotong University between January 2010 and December 2012. During the period of recruitment, each subject was scheduled for an interview after informed consent was written, and a structured questionnaire was administered by the interviewer to collect information about demographic data and risk factors such as smoking status, alcohol use etc. Cervical cancer tissues and tissues adjacent to the tumors were macro-dissected from each subject during operation. In order to ensure a high proportion of tumor cells when collecting tumor tissue, the site and range of tumor were determined and 0.5 m^2^ of tumor tissue outward from the center was captured only with the objects of approximately 1 centimeter in diameter and larger. For 11 normal epithelial cells collection, 0.5 m^2^ of cervix tissue was dissected further than 5 centimeters from the tumor edge and then muscle layer and connective tissue were removed thoroughly to get the high purity of normal cervix epithelia. Within half an hour after tissues dissected, the samples were stored for the DNA methylation and KLF4 expression analysis. The population study was approved by the institutional review board named as “Ethics Committee of Medical School of Xi’an Jiaotong University” in Shannxi, China. Ethics Committee of Medical School of Xi’an Jiaotong University approved the design of cervical cancer study including tissue samples collection.

### Cell Lines and Culture

The human cervical carcinoma cell lines HeLa, SiHa, C33A and CaSki were purchased from the American Type Culture Collection (ATCC). HeLa, SiHa and C33A cells were cultured in Dulbecco’s Modified Eagle’s Medium (DMEM, Sigma-Aldrich, St. Louis, Mo) supplemented with 10% fetal bovine serum (FBS, Invitrogen, Carlsbad, CA) at 37°C in an atmosphere of 5% CO2. CaSki cells were maintained in McCoy’s 5A Medium (Sigma-Aldrich) with 10% FBS.

The human Embryonic Stem Cell (hESC) line H7 (given by the professor Huayan Wang, Department of Animal Biotechnology, College of Veterinary Medicine, Northwest A&F University, Yangling, Shaanxi, China) was obtained the approval from the Ethical Committee of the Xi’an Jiaotong University. Cells were cultured feeder-free in mTeSR medium (Stem Cell Technologies, Vancouver, Canada) on Matrigel (hESC-qualified Matrix, BD Biosciences, CA, USA) in a 5% CO2 normoxic humidified incubator and passaged 1∶6 using accutase solution (Millipore) every 3–7 days.

### 5-Azacytidine Treatment

Cell lines cultured in DMEM with 10% FBS, 24 hours later, the medium was replaced with fresh medium containing 1, 5, or 10 mM 5-Azacytidine (5-Aza; Sigma-Aldrich, Inc, St Louis, MO) or an equal volume of vehicle (PBS). The medium containing drug or vehicle was replaced every 24 hours during a 72-hour period.

### Bisulfite Sequencing and Methylation-Specific PCR

Genomic DNA extraction was performed using the TaKaRa Genomic DNA Extraction Kit (TaKaRa Co., Dalian, China). Genomic DNA (2 ug per sample) was bisulfite modified with the Epitect Bisulfite Kit Protocol (Qiagen), and the modified DNA was amplified using the following primers: BSQ1 forward, 5′-gaaggatttcggttaatttgggg-3′, and reverse, 5′-caaactcgccaaataactacctacg-3′; and BSQ3 forward, 5′-ggttgattatttgaggttaggtgtt-3′, and reverse, 5′-aaaacaattttcaaccaaccatc-3′. The modified DNA was amplified by PCR using 0.2 µM of each primer, 2 units of Hot Start Taq DNA polymerase, and 0.2 mM of each dNTP per reaction. Cycling programs were 95°C for 10 minutes, and then 40 cycles of 95°C for 30 seconds, 54°C for 30 seconds, and 72°C for 30 seconds, followed by a 5-minute incubation at 72°C. The PCR products were examined by gel electrophoresis in 1.5% agarose to confirm that a single band had been obtained and were then sequenced by Invitrogen. Methylation-specific PCR (MS-PCR) was carried out on bisulfate-treated DNA. The primers used were Un-methylated KLF4 forward, 5′-ggttgattatttgaggttaggtgttt-3′, and reverse, 5′-cccaaataacaaaaattacaaacat-3′; and Methylated KLF4 forward, 5′- gttgattatttgaggttaggtgttc-3′, and reverse, 5′-cgaataacgaaaattacaaacgta-3′. Umbilical cord blood DNA was used as a negative control, and it was methylated in vitro by using the Sss1 (CpG) methylase (New England Biolabs).

### Real-time Polymerase Chain Reaction (RT-PCR)

Total RNA was extracted using the Trizol reagent, according to the manufacturer’s protocol (Invitrogen, Carlsbad, CA). 2 ug of total RNA were reverse transcripted using TaKaRa reverse transcriptase (TaKaRa Biotechnology, DaLian, China). A volume of 2.0 ul of each diluted cDNA (1∶20) was subjected to Real-time quantitative PCR in a final volume of 20 ul containing 100 nm of each specific primer and 1×SYBR Green Mix (TaKaRa). The sequences of KLF4 and β-actin primers were as follows: KLF4 gene, F: 5′-aagagttcccatctcaaggcaca-3′, R: 5′-gggcgaatttccatccacag-3′ and β-actin gene, F: 5′-ctaagtcatagtccgcctagaagca-3′, R: 5′tggcacccagcacaatgaa-3′. The amplification was carried out as follows: initial emzyme activation at 95°C for 30 s, then 40 cycles of 95°C for 5 s, 60°C for 30 s, and then a dissociation stage using an iQ5 multicolor real-time PCR Detection System (Bio-RAD, Hercules, CA). The cycle threshold (CT) value was determined as the point at which the fluorescence exceeded a threshold value preset by the instrument’s software. Relative expression of KLF4 in each experiment set (fold-change to control) was calculated according to comparative Ct method using the formula: RQ = 2^−ΔΔCt^.

### Western Blot

Western blot analyses were performed as previously described [22] using cell lysates and an overnight incubation at 4°C with a rabbit polyclonal antibody against human KLF4 (1∶1200 dilution; Santa Cruz Biotechnology) or a mouse monoclonal antibody against human β-actin (1∶500 dilution; Santa Cruz Biotechnology), followed by a secondary incubation using horseradish peroxidaseconjugated anti-rabbit or anti-mouse IgG (Thermo Fisher Scientific Inc., New York, NY). The proteins were briefly incubated with an enhanced chemiluminescence reagent (Millipore, Billerica, Mass) and then visualized on X-ray film.

### Immunocytochemistry

Cells were seeded on coverslips for 48 hours, fixed with 4% paraformaldehyde for 20 minutes, and permeabilized with 0.2% Triton X-100 for 20 minutes at room temperature. The expression of KLF4 in these cells was determined by immunocytochemistry. A standard immunostaining procedure was performed using a rabbit polyclonal antibody against human KLF4 (1∶400 dilutions). We applied the rabbit IgG polyclonal antibody as the isotype control and human embryonic stem cell line H7 as the positive control in ICC. A positive reaction was indicated by a reddish-brown precipitate in the nuclei. KLF4 staining levels in cells were quantified by calculating the percentage of positive cells in ten different visions.

### Cell Growth and Cell Viability Assays

Cells (5×10^4^) were seeded in triplicate in 2-mL media in 6-well plates. The cells were trypsinized and then counted every day for one week using a hemocytometer. A cell growth curve was used to assess the cell proliferation ability. Cell viability was assessed using the 3-(4, 5-dimethylthiazole-yl)-2, 5-diphenyl tetrazolium bromide (MTT; Sigma-Aldrich, St. Louis, Mo) dye according to a standard protocol. The number of viable cells was determined by measuring absorbance at 490 nm.

### Statistical Analysis

Statistical analysis was performed using the SPSS 16.0 software (SPSS Inc, Chicago, IL). The One-way ANOVA analysis was performed to determine the significance of the difference between the covariates. For two groups, independent samples t-test was used to determine statistical significance. To examine the relationship between two quantitative variables, the Pearson’s linear regression analysis was performed. In all the tests, a P<0.05 was defined as statistically significant. Where error bars are presented, they represent ±SEM.

## Results

### The KLF4 Promoter Region is Hypermethylated in Cervical Cancer

**Figure 1:**
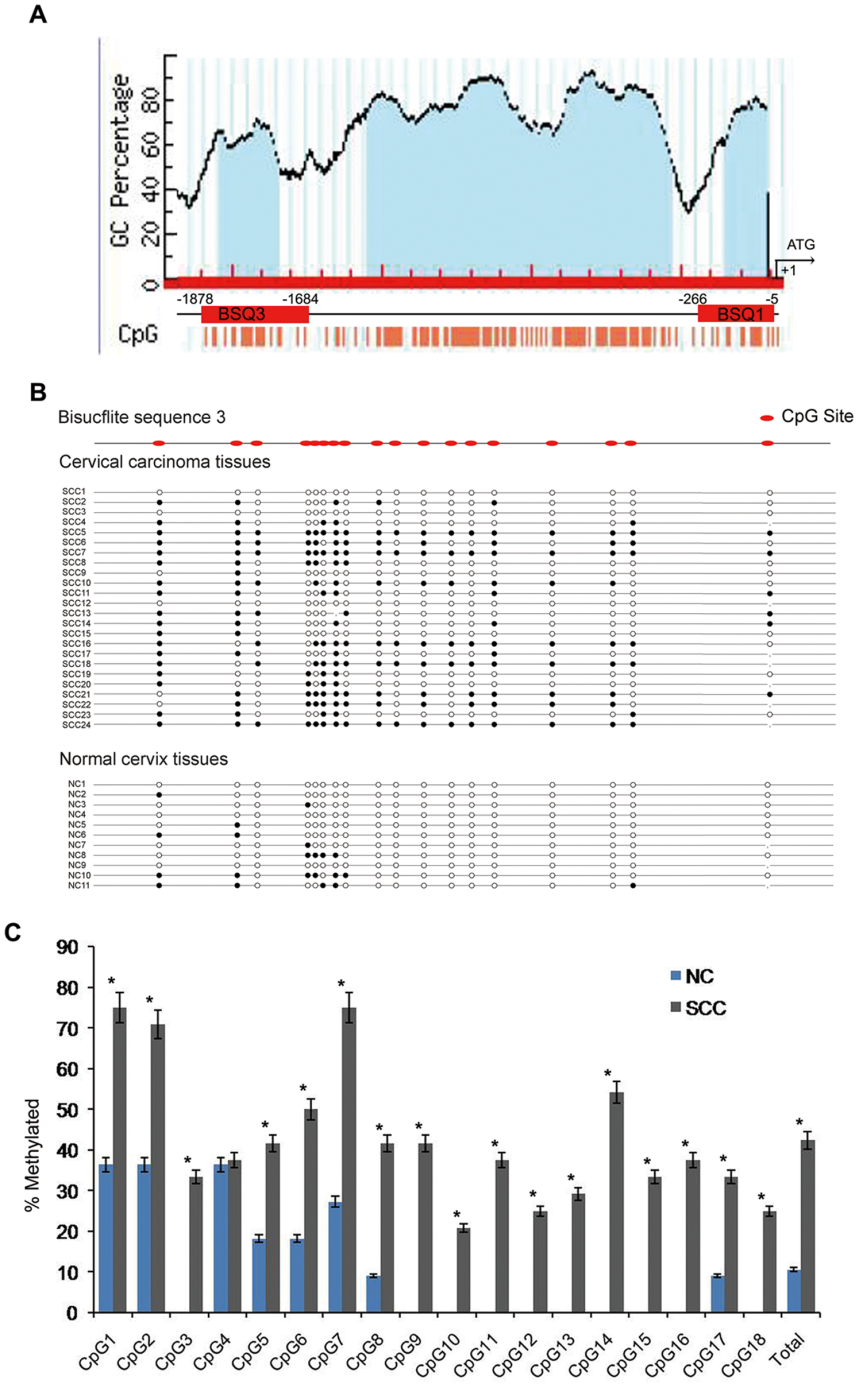
The KLF4 promoter region is hypermethylated in cervical cancer. (A) A schematic representation of the CpG islands found in the promoter region of the KLF4 genomic locus. Numbers indicate positions in bp relative to the transcription start site. The two CpG island regions marked in red were bisulfite sequenced. (B) Bisulfite-converted DNAs from cervical cancer tissues (n = 24) and normal tissues (n = 11) were amplified at the KLF4 promoter, and the fragments were sequenced. CpG sites are represented as boxes, with shaded regions indicating methylation, and unshaded regions indicating no methylation. (C) The methylation level ratio of the CpG sites in cervical cancer and normal cervix tissues. Bars, SE. *, P<0.05.

In a previous study, we demonstrated that KLF4 is downregulated during the development and progression of cervical carcinoma [21]. The overexpression of exogenous KLF4 protein was found to inhibit cervical carcinoma cell growth and tumor formation both *in vitro* and *in vivo* by activating the cell cycle suppressor p27^Kip1^, suggesting that KLF4 works as a tumor suppressor in cervical carcinoma. Promoter CpG island hypermethylation is a common cause in many malignancies, resulting in transcriptional silencing of many tumor suppression genes. The methylation status of the KLF4 promoter was therefore examined in tissues from normal cervix and cervical carcinoma. We profiled two CpG islands upstream of the KLF4 transcriptional start site, from −5 to −266 bp (BSQ1), containing 22 CpG sites, and from −1684 to −1878 bp (BSQ3), containing 18 CpG sites (Fig. 1A). Two pairs of primers were designed to amplify the KLF4 promoter BSQ1 and BSQ3 regions. In the BSQ3 region, we performed quantitative bisulfite sequencing (BSQ) analysis using genomic DNA templates isolated from 24 primary cervical cancer tissues and 12 normal cervix tissues (Fig. 1B). As shown in Fig. 1C, low methylation levels were detected at the KLF4 promoter BSQ3 region in normal cervix samples (average methylation level was 11.11%). However, in cervical cancer tissues, methylation levels in this region were significantly higher than in normal cervix tissues at each individual CpG site except CpG4 (41.9%, P<0.05). In the BSQ1 region of the KLF4 promoter, low methylation levels were detected in both cervical cancer and normal cervix tissues (data not shown). Altogether, these results suggest that hypermethylation of the KLF4 promoter BSQ3 region, and not the BSQ1 region, is involved in cervical carcinogenesis.

### KLF4 Promoter Methylation Negatively Correlates with Gene Expression at Both the Transcriptional and the Translational Levels

**Figure 2:**
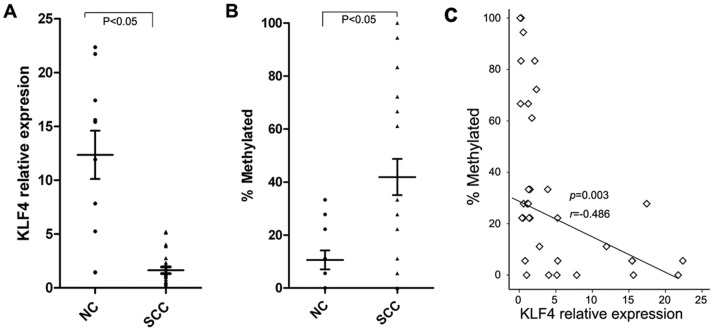
KLF4 promoter methylation is negatively correlated with the gene expression. (A) The relative mRNA levels of KLF4 in cervical cancer and normal cervix tissues. (B) The methylation ratio of the KLF4 promoter BSQ3 region. (C) An inverse correlation is observed between KLF4 promoter methylation and mRNA levels (r = −0.486, P = 0.003).

KLF4 transcriptional levels were determined in these 24 cervical carcinoma and 12 normal cervix samples by Real-time PCR. The average relative expression (compared with GAPDH) of KLF4 was 12.36±2.24 in normal cervix but 1.642±0.31 in cervical carcinoma (Fig. 2A). Normal cervix tissues expressed 6.54 times more KLF4 mRNA than cervical carcinomas did (P<0.05). The methylation assay results for the KLF4 promoter BSQ3 region in these 24 cervical carcinoma and 12 normal cervix samples are summarized in Fig. 2B and Table 1. In cervical carcinomas, the methylation level is more than 3-times higher than in normal cervix. Correlation analysis showed that the KLF4 promoter methylation status was inversely related to KLF4 expression at the transcriptional levels in both cervical carcinoma and normal cervix tissues (Fig. 2C, P = 0.003, r = −0.486).

**Table 1:** Correlation of methylation status of KLF4 gene and the protein expression in SCC and NC.

Sample	KLF4 IHC(score)	KLF4methylation (%)	P Value(chi square)
SCC(24)	2.45±2.94	41.90%	
NC(11)	9.30±2.85	11.11%	<0.05

KLF4 protein expression in cervical carcinoma and normal cervix specimens was detected by immunohistochemistry, quantified as the IHC score and summarized in Table 1. The average IHC score of KLF4 staining was 9.30±2.85 in normal cervix and 2.45±2.94 in invasive carcinoma (P<0.05 by 2-tailed *t* test). Normal cervical tissues expressed more than three times KLF4 protein than invasive cervical carcinomas did. KLF4 protein expression and promoter methylation also showed significant negative correlation in both cervical carcinoma and normal cervical tissues (P<0.05, Table 1), suggesting that KLF4 inactivation at the transcriptional level may attribute to its suppression at the protein level. When the cancer samples were grouped according to their clinical pathological features, the KLF4 methylation status did not correlate with the histological grade, clinical stage, or lymphatic metastasis age of the patients (P>0.05, Table S1). We conclude that this study sample is too small for correlating the KLF4 promoter methylation state with clinical features. Together, these results suggest that KLF4 inactivation in cervical carcinomas results from its promoter methylation.

### Methylation of the KLF4 Promoter in Cervical Cancer Cell Lines

**Figure 3:**
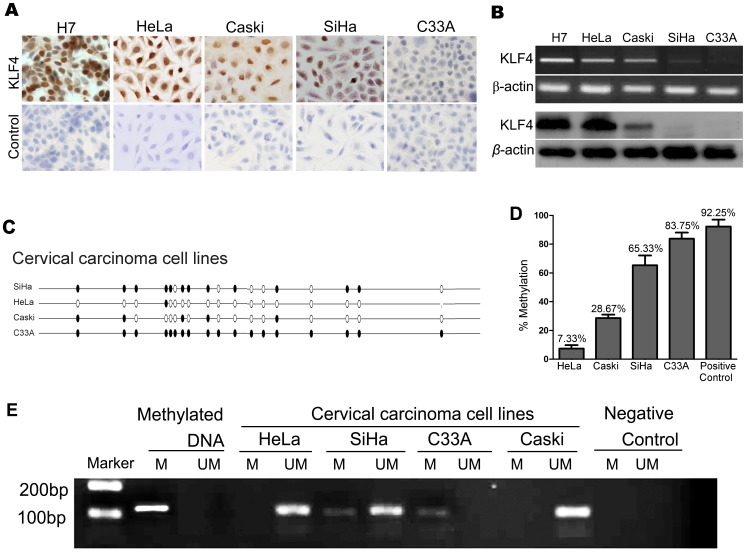
Methylation of the KLF4 promoter in cervical cancer cell lines. (A and B) KLF4 expression in the 4 cervical cancer cell-lines HeLa, CaSki, SiHa and C33A was detected by IHC (A) and by PCR and western blot (B). We applied the human embryonic stem cell line H7 as a positive control and the rabbit IgG polyclonal antibody as the isotype control in immunocytochemistry. (C) Bisulfite sequencing of the KLF4 promoter in cervical cancer cell-lines. (D) Statistical analysis of KLF4 promoter methylation in cervical cancer cell-lines. (E) MS-PCR for a region of the KLF4 promoter in the 4 cervical cancer cell lines. A methylated band was amplified in SiHa and C33A cells. Globally methylated DNA from normal fetal cord blood samples was included as a positive control for the methylated (M) and unmethylated (U) primers.

As shown in Fig. 3A, with immunocytochemical assays, the KLF4 protein was found to be strongly expressed in HeLa and CaSki cells and weakly expressed in SiHa cells, but it was barely expressed in C33A cells. RT-PCR and western blot analyses further confirmed the expression results in these four cell lines at the transcriptional and translational levels, respectively (Fig. 3B). We applied the human embryonic stem cell line H7 as a positive control and the rabbit IgG polyclonal antibody as the isotype control in immunocytochemistry.

The CpG methylation status of the KLF4 promoter was determined by BSQ sequencing in the four cell lines (Fig. 3C). Approximately 65.33% and 83.75% methylation levels were found in SiHa and C33A cells, respectively, but only approximately 28.67% methylation was observed in Caski cells, and extremely rare methylation (∼7.33%) was detected in HeLa cells. These data are summarized in Figure 3D. To confirm the reliability of the methylation sequencing method, methylation-specific PCR (MS-PCR) was performed on the CpG1 site of BSQ3 (position −1875) (Fig. 3E). The methylation levels detected by the MS-PCR assay were consistent with those of the BSQ method in the four cervical cancer cell lines. HeLa and CaSki cells exhibited low levels of promoter methylation and higher levels of KLF4 protein and mRNA; conversely, SiHa and C33A cells showed high levels of promoter methylation and little to no KLF4 mRNA and protein. These results suggest that KLF4 expression in cervical carcinoma cell lines is negatively correlated with the promoter methylation level.

### KLF4 Expression at the Transcriptional and the Translational Levels is Drastically Enhanced by 5-Aza Treatment

To further confirm the role of promoter methylation in the transcriptional regulation of the KLF4 gene, SiHa and C33A cells, in which the KLF4 promoter was heavily methylated, were treated with the demethylating agent 5-Aza; this agent causes DNA demethylation via inhibition of DNA methyltransferase activity. After treatment with different doses of 5-Aza for 72 hours, KLF4 promoter methylation was examined by BSQ3 sequencing, and KLF4 expression was assayed at the transcriptional level by the Real-time PCR and at the translational level by western blot analysis.

**Figure 4:**
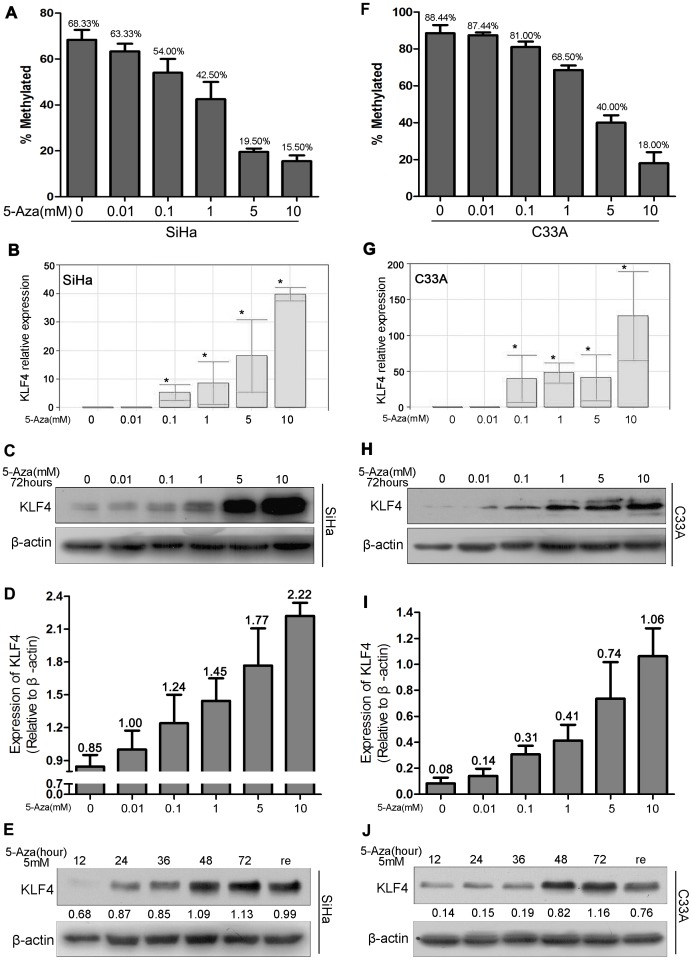
KLF4 expressions at both the transcriptional and the translational levels is drastically enhanced after treatment with the demethylating agent 5-Azacytidine. (A) Bisulfite sequencing of the KLF4 promoter in SiHa cells after treatment with different doses of 5-Aza. (B) KLF4 mRNA levels were quantified by PCR for three independent RNA samples from SiHa cells after treatment with different doses of 5-Aza, *, P<0.05. (C) KLF4 protein expression in SiHa cells was gradually enhanced in response to increasing doses of 5-Aza. (D) The relative expression of KLF4 protein in SiHa cells treated with different doses of 5-Aza. (E) KLF4 protein expression in SiHa cells was gradually enhanced during the time-course of treatment with 5 mM 5-Aza; it was reduced upon 5-Aza withdrawal following a 72-hour treatment. (F) Bisulfite sequencing of the KLF4 promoter in C33A cells after treatment with different doses of 5-Aza. (G and H) KLF4 expression was detected by PCR and western blot in C33A cells treated with different doses of 5-Aza in three independent repeats, *, P<0.05. (I) The relative expression of KLF4 protein in C33A cells treated with different doses of 5-Aza. (J) KLF4 protein expression was monitored during the time-course of treatment with 5 mM 5-Aza and during agent withdrawal following a 72-hour treatment. The relative levels of KLF4 protein normalized to β-actin are shown. Bars indicate SE. *, P<0.05.

In SiHa cells, treatment with 0.00, 0.01, 0.10, 1.00, 5.00 and 10.00 mM of 5-Aza resulted in a gradual decrease in KLF4 promoter methylation levels from 68.33% to 15.50% (Fig. 4A, P<0.05). At the same time, the relative expression of KLF4 gradually increased from 1±0.37 to 40±1.98 at the transcriptional level (Fig. 4B, P<0.05) and from 0.85 to 2.22 at the translational level (Fig. 4C and D, P<0.05). Similarly, in C33A cells, KLF4 promoter methylation levels gradually decreased from 88.44% to 18.00% (Fig. 4F, P<0.05), and the relative expression of KLF4 gradually increased from 1±0.32 to 134±56.82 at the transcriptional level (Fig. 4G, P<0.05) and from 0.08 to 1.06 at the translational level (Fig. 4H and I, P<0.05) after a 72-hour treatment with 5-Aza (0.00, 0.01, 0.10, 1.00, 5.00 and 10.00 mM). These results indicate that promoter hypermethylation is the main cause for KLF4 inactivation in these two cervical carcinoma cell lines. Furthermore, when SiHa and C33A cells were treated with 5 mM of 5-Aza for 12, 24, 48, and 74 hours, the relative protein levels of KLF4 gradually increased from 0.68 to 1.13 in SiHa cells (Fig. 4E, P<0.05) and from 0.14 to 1.16 in C33A cells (Fig. 4J, P<0.05) throughout the treatment time-course. After 72 hours of 5-Aza treatments, 5-Aza was washed off, and the cells were continuously cultured for another 48 hours without 5-Aza; this caused a decrease in KLF4 protein levels from 1.13 to 0.99 in SiHa cells (Fig. 4E, P<0.05) and from 1.16 to 0.76 in C33A cells (Fig. 4J, P<0.05). These results indicate that the 5-Aza demethylating activity is a dynamic process and further support the notion that promoter hypermethylation is the main cause for KLF4 inactivation in the cervical carcinoma cell lines SiHa and C33A.

### Restored Expression of KLF4 by 5-Aza Inhibits the Proliferation and Increased the Chemosensitivity for Cisplatin in Cervical Cancer Cells

**Figure 5:**
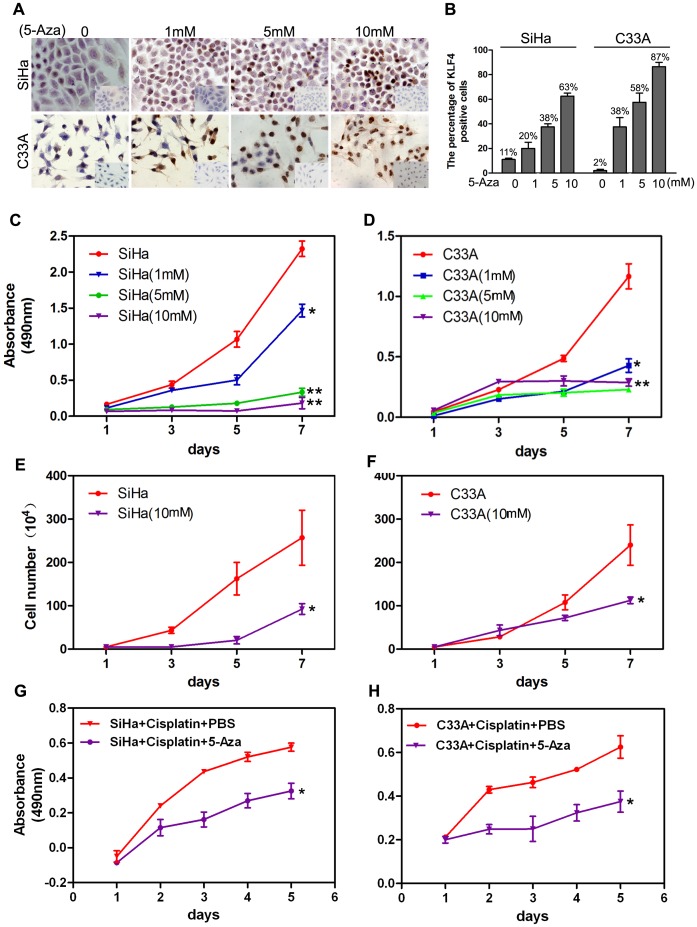
Restored expression of KLF4 by 5-Aza inhibits the proliferation of cervical cancer cells. (A and B) KLF4 protein expression gradually increased in response to different doses of 5-Aza in SiHa and C33A cells, as detected by ICC. (C and D) The proliferation of SiHa (C) and C33A (D) cells treated with different doses of 5-Aza was determined by counting cells longitudinally. (E and F) The viability of SiHa and C33A cells treated with 10 mM 5-Aza was determined by the MTT assay. (G and H) The cell survival rate of cervical cancer cell lines SiHa and C33A treated by chemistry agent cisplatin was detected by the MTT assay. Bars indicate SE. *, P<0.05.

We previously showed that overexpression of KLF4 results in the retardation of cell growth and tumor formation in cervical cancer cells [21]. Here, increasing doses of 5-Aza treatments gradually augmented KLF4 protein levels, as determined by IHC from 11% to 63% in SiHa cells and 2% to 87% in C33A cells (Fig. 5A and B, P<0.05). The proliferative ability of SiHa (Fig. 5C and E) and C33A (Fig. 5D and F) cells was significantly suppressed, as shown by MTT assays (P<0.05, Fig. 5C and D) and by cell growth curve analysis (P<0.05, Fig. 5E and F). In addition, when cervical cancer cell line SiHa and C33A were treated with 50 ug/ml chemistry agent cisplatin, the cell survival rate was much lower in the present of 5-Aza than that in PBS (P<0.05, Fig. 5G and H). These results imply that KLF4 inactivation significant inhibited the cell proliferation and increased the chemosensitivity for cisplatin in cervical cancer cells, although 5-Aza is not a specific KLF4 demethylation agent.

## Discussion

Epigenetic gene silencing through DNA methylation has been suggested to be one of the important steps in cervical carcinogenesis. Promoter hypermethylation of P16, DKAP, CDH1 and other related tumor suppressor genes was linked to clinical pathological parameters in cervical cancer [23], [24], [25], [26]. In contrast, methylated carcinogenic HPV DNA was a predictive and/or diagnostic biomarker for risk of cervical cancer among HPV-positive women [27], [28].

KLF4 has been shown to interact with a number of pathways with well-documented links to cervical cancer biology. KLF4 transactivates the expression of the cell cycle inhibitor p27^Kip^, which is associated with malignant transformation and aggressive phenotypes of cervical neoplasms [29], [30]. KLF4 represses the Wnt signaling pathway, which was shown to be hyperactivated in a subset of cervical cancer [31], [32], [33]. Notch signaling represses KLF4 in the gastrointestinal tract [34], [35]. Epithelial transformation by KLF4 requires Notch1 but not canonical Notch1 signaling, and Notch signaling plays an important role in the development and progression of cervical cancer [36], [37], [38], [39]. This result prompted us to further explore the mechanism of action of KLF4 in cervical cancer.

Here, we determined that KLF4 promoter methylation was 4-fold higher in cancer samples and also markedly higher in some cervical cancer cell lines, compared with control samples. KLF4 expression was inversely related to methylation status. Moreover, the expression of KLF4 protein and mRNA was restored upon treatment of cervical cancer cell lines with 5-Aza, which inhibited the cell proliferation and increased the chemosensitivity for cisplatin. These findings indicate that promoter methylation suppresses KLF4 gene transcription and thus contributes to inactivating KLF4’s tumor suppressor function in cervical carcinogenesis.

Although mutation of the KLF4 gene was shown to cause a defect in the proliferation and differentiation of gastric mucosal epithelium, it was concluded that a genetic alteration of the KLF4 gene might play a minor role in gastric carcinogenesis [19]. KLF4 is inactivated by either genetic or epigenetic mechanisms in a large subset of medulloblastomas, and it likely functions as a tumor suppressor gene in the pathogenesis of medulloblastoma [12]. Interestingly, the hypermethylation pattern of the KLF4 promoter region was variable among several types of tumors. In gastric cancer, KLF4 promoter methylation was reported in the −156 to −39 bp region relative to the ATG [19], [40]. A methylated CpG island in the −2154 to −1796 bp region of the KLF4 promoter was detected in medulloblastoma [12]. In the present study, we assayed the methylation status in the two regions of the KLF4 promoter, and our results suggest that the −1684 to −1878 bp region is hypermethylated in cervical cancer. However, the region near the ATG (−5 to −266 bp) was rarely methylated in either cervical cancer or normal cervix samples. The methylation of the KLF4 promoter region in cervical cancer was different from that of other type of tumors. Further studies should focus on identifying the key region influencing KLF4 gene expression, by using KLF4 genome-wide methylation scanning.

In summary, by using the BSQ technology, we uncovered a change in the methylation status of the KLF4 gene in cervical cancer. KLF4 methylation levels were inversely correlated with the gene’s transcription, and KLF4 expression was restored upon treated with the demethylating agent 5-Aza. The restored KLF4 expression inhibited the cervical cancer cell survival in the treatment of cisplatin. We conclude that the promoter hypermethylation of KLF4 inactivates its function as a tumor suppressor in cervical carcinogenesis.

## Supporting Information

Table S1 **Correlation of KLF4 methylation status with clinic pathological features such as age, Grade, Stage and Lymph node in cervical cancer.**
(DOC)
