# Supplementary material for: Promoter Hypermethylation of KLF4 Inactivates Its Tumor Suppressor Function in Cervical Carcinogenesis
Source: PLoS One. 2014 Feb 14;9(2):e88827. doi: 10.1371/journal.pone.0088827 (PMC3925171; doi:10.1371/journal.pone.0088827)
Supplement: Table S1 — Correlation of KLF4 methylation status with clinic pathological features such as age, Grade, Stage and Lymph node in cervical cancer. (DOC) [file pone.0088827.s001.doc]

| **Table S1. Correlation of KLF4 methylation status with clinic pathological features in cervical cancer** | | | | |
| --- | --- | --- | --- | --- |
| Clinic pathological | n | Methylation (%) | Un-methylation (%) | P Value |
| Age |  |  |  |  |
| <45 | 12 | 41.67% | 58.33% | 0.833 |
| ≥45 | 12 | 42.13% | 57.87% |
| Grade |  |  |  |  |
| Low | 6 | 40.74% | 59.26% | 0.340 |
| moderately | 10 | 37.78% | 62.22% |
| High | 8 | 47.92% | 52.08% |
| Stage |  |  |  |  |
| I | 11 | 36.36% | 63.64% | 0.552 |
| II | 8 | 46.90% | 53.10% |
| III-IV | 5 | 45.37% | 54.63% |
| Lymph node |  |  |  |  |
| Yes | 4 | 47.22% | 52.78% | 0.238 |
| No | 20 | 40.83% | 59.17% |
